# Highly Efficient Electrospun Silver Decorated Graphene Oxide Nanocomposites on Poly(vinylidene fluoride) (PVDF@GO-Ag) Hybrid Membrane for Reduction of 4-Nitrophenol

**DOI:** 10.3390/molecules29163930

**Published:** 2024-08-20

**Authors:** Xiaoben Yang, Zhen He, Lei Jin, Huiyang Chen, Qianglin Li, Ling Wu, Zhenghong Huang, Mingxi Wang

**Affiliations:** 1 Key Laboratory of Biomass-Based Materials for Environment and Energy in Petroleum & Chemical Industries, School of Chemical Engineering and Pharmacy, School of Chemical and Environmental Engineering, Wuhan Institute of Technology, Wuhan 430205, China; little_ben2002@163.com (X.Y.); hezhenwork@126.com (Z.H.); 17371087162@163.com (L.J.); 18154351008@163.com (H.C.); 2 Department of Material and Environmental Engineering, Chengdu Technological University, Chengdu 611730, China; 3 Hubei Province Key Laboratory of Coal Conversion and New Carbon Materials, School of Chemistry and Chemical Engineering, Wuhan University of Science and Technology, Wuhan 430081, China; wuling2018@wust.edu.cn; 4 Key Laboratory of Advanced Materials Ministry of Education, School of Materials Science and Engineering, Tsinghua University, Beijing 100084, China; zhhuang@tsinghua.edu.cn

**Keywords:** graphene oxide-sliver, poly(vinylidene fluoride) hybrid membrane, electrospun, 4-nitrophenol, catalytic reduction

## Abstract

Graphene oxide-silver poly(vinylidene fluoride) membranes (PVDF@GO-Ag) were successfully synthesized by the electrospinning method, which exhibited a high catalytic activity using the hydrogenation of 4-nitrophenol (4-NP) as a model reaction in a batch reaction study. The hybrid membranes doped with 1 wt% GO and 2 wt% Ag (PVDF-1-2) exhibited the most desired performance for the catalytic reduction of 4-NP. Importantly, PVDF-1-2 exhibited excellent cycling stability in 10 catalytic cycle tests and was highly amenable to separation. This property effectively addresses the significant challenges associated with the practical application of nanocatalysts. Furthermore, density-functional theory (DFT) calculations have demonstrated that the GO-Ag nanocomposites exhibit the strongest adsorption capacity for 4-NP^−^ when a specific ratio of GO and Ag is achieved, accompanied by the loading of Ag nanoclusters onto GO. Additionally, the study demonstrated that an increase in temperature significantly accelerated the reaction rate, in line with the van’t Hoff rule. This study provides an effective and environmentally friendly solution for the treatment of 4-NP in wastewater.

## 1. Introduction

As a kind of aromatic compound, nitrophenols are regarded as most prevalent organic contaminants in wastewater discharged from agricultural and industrial sources, since nitrophenols are key reagents in the production of pesticides, plasticizers, fungicides, and dyes, as well as other products [1,2,3]. In particular, 4-nitrophenol (4-NP), one of the representative nitrophenols, has been reported as a priority pollutant by US Environmental Protection Agency [4] due to its high toxicity and carcinogenicity [5,6]. The residue of 4-NP in wastewater jeopardizes the ecological environment and human health, so it is a matter of urgency to remove it from water with an effective and environmentally friendly method. However, it a challenge to eliminate 4-NP from wastewater because it is highly soluble and very stable in water [7].

Numerous efforts have been contributed towards the treatment of 4-NP; many methods including adsorption, advanced oxidation, biological and microbial degradation, chemical catalytic reduction, and electrochemical methods have been explored to remove 4-NP from water [8,9,10,11]. Among these methods, catalytic reduction of 4-NP into 4-aminophenol (4-AP) by sodium borohydride (NaBH_4_) is the most effective and environmentally friendly method for the degradation of 4-NP [12,13] because not only is the reduction process highly conversional and time-saving but the reduction product is also value-added, with less toxicity and a wide range of applications. 4-AP is an important intermediate for the production of antipyretic and analgesic drugs [14], as well as pharmaceutical and plastic products [15], and catalytic reduction is a promising strategy to treat 4-NP in water. 

It is crucial to develop effective catalysts for achieving efficient conversion of 4-NP into 4-AP. Various nanostructures have been fabricated and employed as catalysts for the reduction of nitrophenols in the presence of reducing agents [16], for example, noble metals, transition metal oxides, metal-free carbon, and their composites. Nanostructured noble metal nanoparticles (NPs) are desirable catalysts for 4-NP reduction due to their outstanding catalytic performance and high efficiency. Almost all the noble metals (i.e., Pd, Rh, Au, Pt, etc.) have been studied to catalytically reduce 4-NP [17,18], showing satisfactory catalytic activity. However, they are seldom employed in industrial applications because they are scarce and quite expensive, except for silver. As a consequence, Ag NPs are the most widely investigated catalysts for 4-NP reduction. Unfortunately, naked Ag NPs are highly susceptible to aggregation and oxidation during catalytic reactions owing to their high surface energy [15], resulting in a decrease in the catalytic efficiency. Many approaches have been applied to overcome this problem. Immobilizing NPs on catalytic supports is a feasible strategy, with examples such as cellulose nanofibers [19], carbon materials [20], graphene oxides (GO) composites, and polymeric matrices. 

Among these supports, GO is one of the most popular. GO is a newly emerging two-dimensional material, which has some unique merits that have led to its use in many fields over the past few years. Firstly, it contains a number of oxygenated groups on their surface, such as carboxyl, carbonyl, hydroxyl, and epoxy, and the presence of oxygen-containing functional groups render it compatible with most of the organic polymers [21]. Secondly, GO has a large theoretical specific surface area (2630 m^2^/g), which can create an appropriate platform for the stabilization of nanoparticles (NPs) acting as catalysts. For example, GO has been applied as a desirable support to anchor gold NPs for catalysis and silver NPs for antibacterial applications [22,23]. Thirdly, GO has other advantages, i.e., good water solubility, low cytotoxicity and high electrical properties [24]. On account of these characteristics, GO has been successfully utilized as a support to disperse and stabilize metal NPs. For example, Au/GO heterostructures were prepared and used for the reduction of 4-NP [25], and the Au/GO nanocomposites showed much better catalytic activity than that of pristine Au NPs. Besides Au, silver (Ag) nano metal particles have been turned into potential materials for applications in catalysis, antibacterial, and antifungal roles; Ag NPs anchored on GO (GO-Ag) have good catalytic ability in the reduction of 4-NP [26,27,28]. Although studies have demonstrated that the supported metal catalysts yield higher catalytic activity than bare catalysts due to less aggregation, there are still difficulties in reusability inherited from their particle form. In practical applications, reusability is a crucial parameter that should be considered when choosing a proper catalyst for any reaction, so the catalysts must be easily recycled and reused. However, the recycling of GO-Ag nanocomposites used in aqueous solution usually undergoes resource-intensive and time-consuming processes, which includes centrifugation, washing, and redispersion cycles, hampering the reusability of the recycled catalysts. Combining the supported metal catalysts with membrane can effectively solve the above problem. 

In recent years, catalytic membrane reactors (CMRs) have attracted tremendous interest because of their incorporation of catalysis and separation processes into one unit subtly [29]. Moreover, CMRs are considered to be effective for the treatment of industrial wastewater owing to their mild operation, high selectivity, easy amplification, and low energy consumption and operating investment [30,31]. The traditional method for immobilizing catalytic metal NPs is primarily direct deposition on the surface of a polymeric membrane [32,33], which suffers from the leaching of metal NPs immobilized on the membrane surface due to the weak binding of metal NPs to the inert interface [34]. Electrospinning is a versatile process used to produce polymer membranes, and electrospun polymer membranes in general exhibit high porosity with interconnected open pores. More importantly, the catalysts can be directly incorporated into the membrane matrix by a one-step electrospinning method with strong binding between them, leading to rare leaching of metal NPs. 

On the basis of the above facts, we have endeavored to develop new hybrid nanocomposites membranes containing GO-Ag as catalytic active components for application in the reduction of 4-NP. The hybrid nanocomposites membranes were fabricated by the electrospinning method, obtaining well-dispersed Ag NPs on GO within a poly(vinylidene fluoride) (PVDF) membrane (PVDF@GO-Ag), and their catalytic potential for 4-NP reduction was investigated. The as-synthesized composite membranes exhibited efficient catalytic activity for the reduction of 4-NP to 4-AP.

## 2. Results and Discussions

### 2.1. Characterization of PVDF@GO-Ag Hybrid Membranes

**Figure 1:**
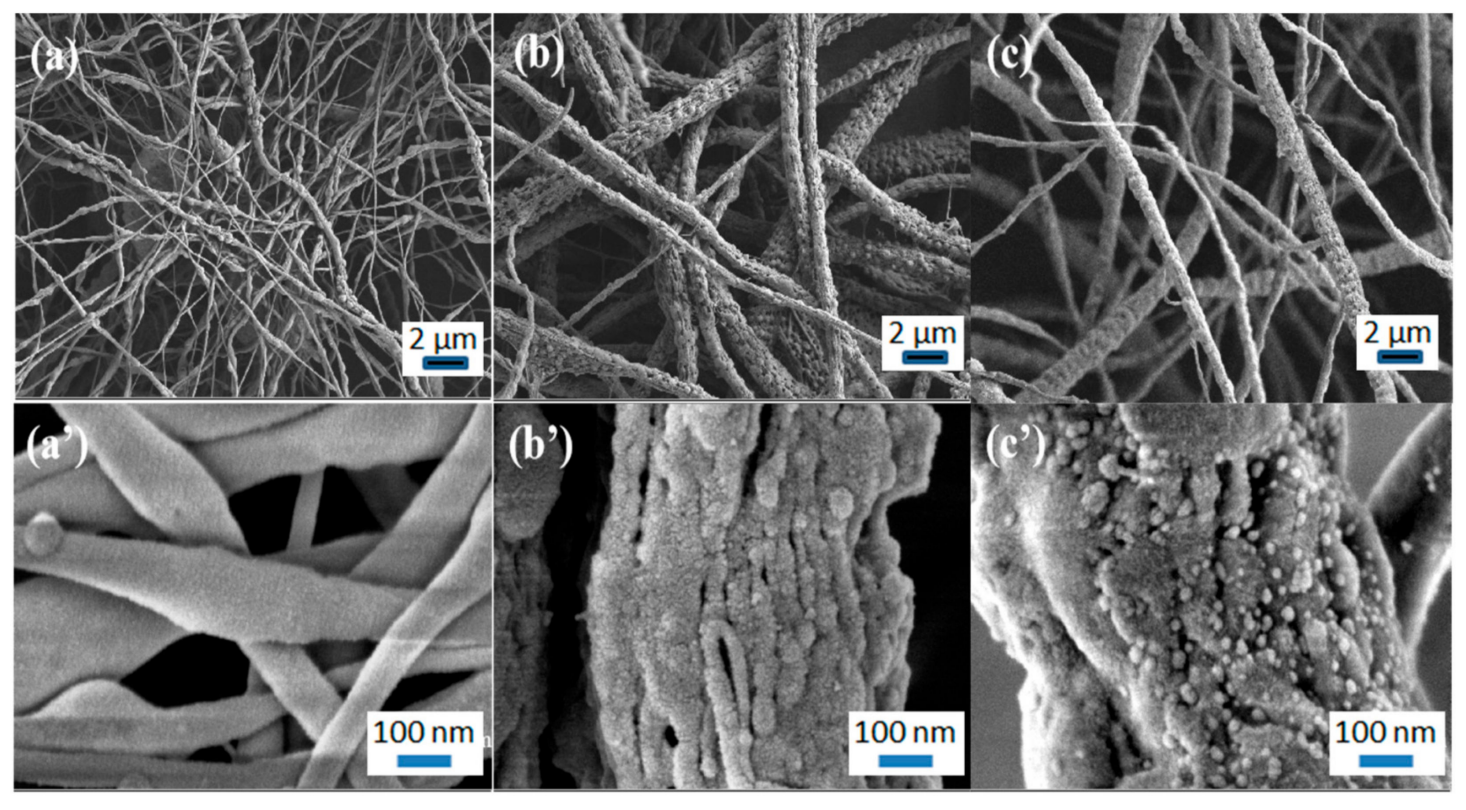
SEM images of (**a**,**a’**) PVDF, (**b**,**b’**) PVDF-0-2, and (**c**,**c’**) PVDF-1-2 fibrous membranes.

The representative SEM images in Figure 1 disclose the surface morphology and structure of the three PVDF-based fibrous membranes. It was easily observed from Figure 1a,a’ that a number of beaded nanofibers are formed in the pristine PVDF nanofibrous layer with inhomogeneous diameter fibers. The formation of beads is correlated with the saturated vapor pressure of the solvents, the electrical conductivity of the solute, and PVDF concentration and injection rate during electrospinning process [35], and a higher PVDF concentration and lower injection rate will inhibit the bead formation. The special structure in which the membrane surface is covered with bead-fibers can hold the water droplets on it, leading to improved hydrophobicity of the membrane [36], which can be confirmed by the contact angle measurements. After the addition of AgNO_3_, the electrospun fibrous membrane in Figure 1b,b’ shows thicker fibers with much fewer beads due to the enhanced conductivity of the mixed spinning solution, and the fibers are uniformly distributed. Meanwhile, the fibers become rough and some very small granular particles are attached to the fiber surface, indicating that Ag NPs have been successfully doped into the fibers. It is noteworthy that irregular and narrow pores were observed on the surface of both PVDF-0-2 and PVDF-1-2 fibers. This phenomenon can be attributed to the fact that the boiling point of dimethylamine produced during the reduction of silver ions is lower than that of DMF, resulting in its volatilization before DMF and the formation of pore structures. Figure 1c,c’ displays the SEM images of PVDF-1-2 fibrous membranes. With the introduction of GO, the fiber diameter of the hybrid membrane became thicker because GO can further improve the conductivity of the spinning solution. More importantly, there are a large number of oxygen radicals with negative charges on the GO surface, which can attract Ag^+^ ions, providing nucleation sites of Ag so that more Ag NPs could be decorated on the membrane and the catalytic performance can thus be greatly improved. Furthermore, the presence of a considerable number of minute particles on the fiber surface indicates that the GO-Ag nanocomposites were successfully loaded onto the fibers. 

**Figure 2:**
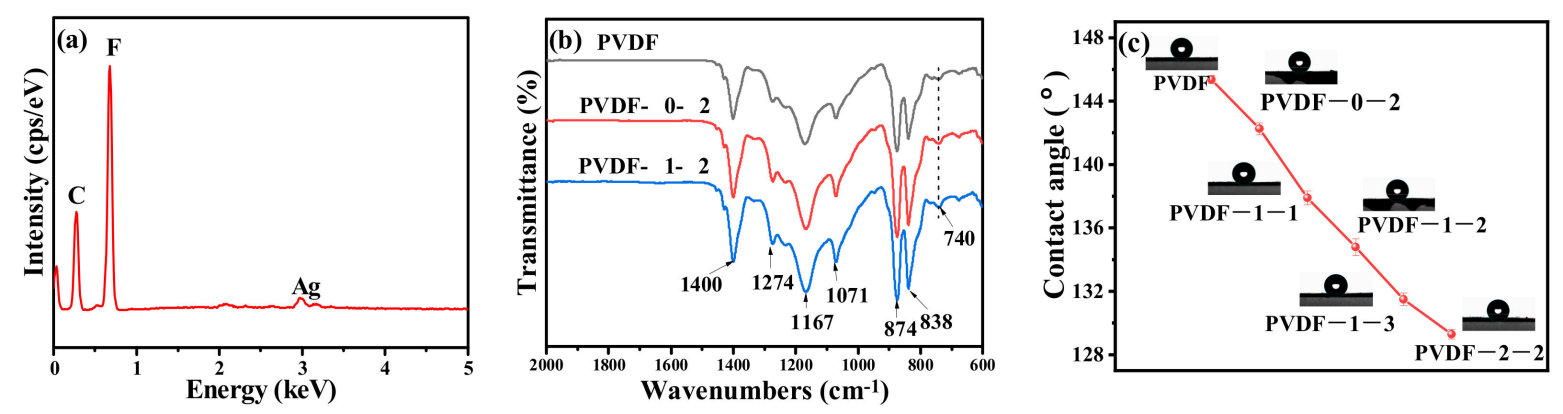
(**a**) EDS analysis of PVDF-1-2; (**b**) FTIR spectra of PVDF, PVDF-0-2, and PVDF-1-2. (**c**) Water contact angle test of the electrospun pristine and hybrid PVDF−based fibrous membranes.

To examine the incorporation of Ag into the prepared membranes, energy-dispersive spectroscopy (EDS) analysis was conducted on the PVDF-1-2. Figure 2a presents the new elemental Ag and O spectra, indicative of the successful decoration of Ag and nanocomposites GO-Ag in the hybrid membrane. In order to further verify the presence of GO-Ag in the PVDF matrix membrane, the FTIR spectra of pristine PVDF, PVDF-0-2, and PVDF-1-2 were recorded. As shown in Figure 2b, similar characteristic peaks are observed for all the spectra in the wave number range of 600~1600 cm^−1^. The peaks located at 1400, 1274, and 1167 cm^−1^ are correlated with the CH_2_ deformation vibrations and CF and CF_2_ stretching vibrations of PVDF [37,38]. The peaks at 740 and 1071 cm^−1^ correspond to the α-phase, and the peaks at 838, 874, and 1274 cm^−1^ are ascribed to the β-phase of PVDF [39]. With the addition of GO, Ag, or both GO and Ag to PVDF, the intensities of α-phase peaks decreased while those of β-phase peaks increased concomitantly. It is recognized that the strong interaction between carbonyl groups in GO and fluorine groups in PVDF causes a transformation of the α-phase’s trans-gauche-trans-gauche conformation into the trans-trans conformation of the β-phase [40]. This transformation verified that GO, Ag, or both of them had been successfully introduced into the PVDF matrix.

For industrial applications in practice, the membrane with improved hydrophilicity is conducive to preventing fouling caused by organic contaminants. Water contact angle (WCA) measurement is a powerful technique for evaluating the wettability of fibrous membranes. The photographs of water droplets on all the electrospun PVDF-based membrane surfaces are shown in Figure 2c. The pristine PVDF fibrous membrane presents a high contact angle with value of 145.3 ± 0.3°, suggesting its hydrophobic characteristic. With the addition of 2 wt% Ag into PVDF, the contact angle with water of PVDF-0-2 membrane slightly decreased to 142.4 ± 0.4°. As the Ag and GO were added into PVDF simultaneously, the WCAs of the as-electrospun hybrid membrane dropped more pronouncedly. When Ag content relative to PVDF was 1, 2, and 3 wt% while the GO content was fixed at 1 wt%, the WCAs of them were 137.8 ± 0.4°, 134.7 ± 0.5°, and 131.5 ± 0.4°, respectively, which are much less than those of pristine PVDF and PVDF-0-2. Obviously, the incorporation of Ag NPs into PVDF matrix led to the decrease in WCA, indicating the improved hydrophilicity of the PVDF-based membranes. The enhanced hydrophilicity of the composite membrane may be attributed to the uniform dispersion of Ag NPs on the PVDF surface, which increases the surface roughness and provides additional hydrophilic sites, thereby reducing the WCA further [41]. Moreover, the WCA was further reduced to 129.3 ± 0.3° by hybridizing 2 wt% GO with 2 wt% Ag in PVDF, so the PVDF-2-2 hybrid membrane was found to display the lowest WCA. GO contains a substantial number of oxygen-containing functional groups which are capable of forming hydrogen bonds with water molecules, thereby enhancing the hydrophilic character of the material. The addition of GO and Ag NPs to PVDF results in a further reduction in WCA, which may be attributed to the additional hydrophilic functional groups of GO and its high specific surface area, facilitating the adsorption of water molecules.

**Figure 3:**
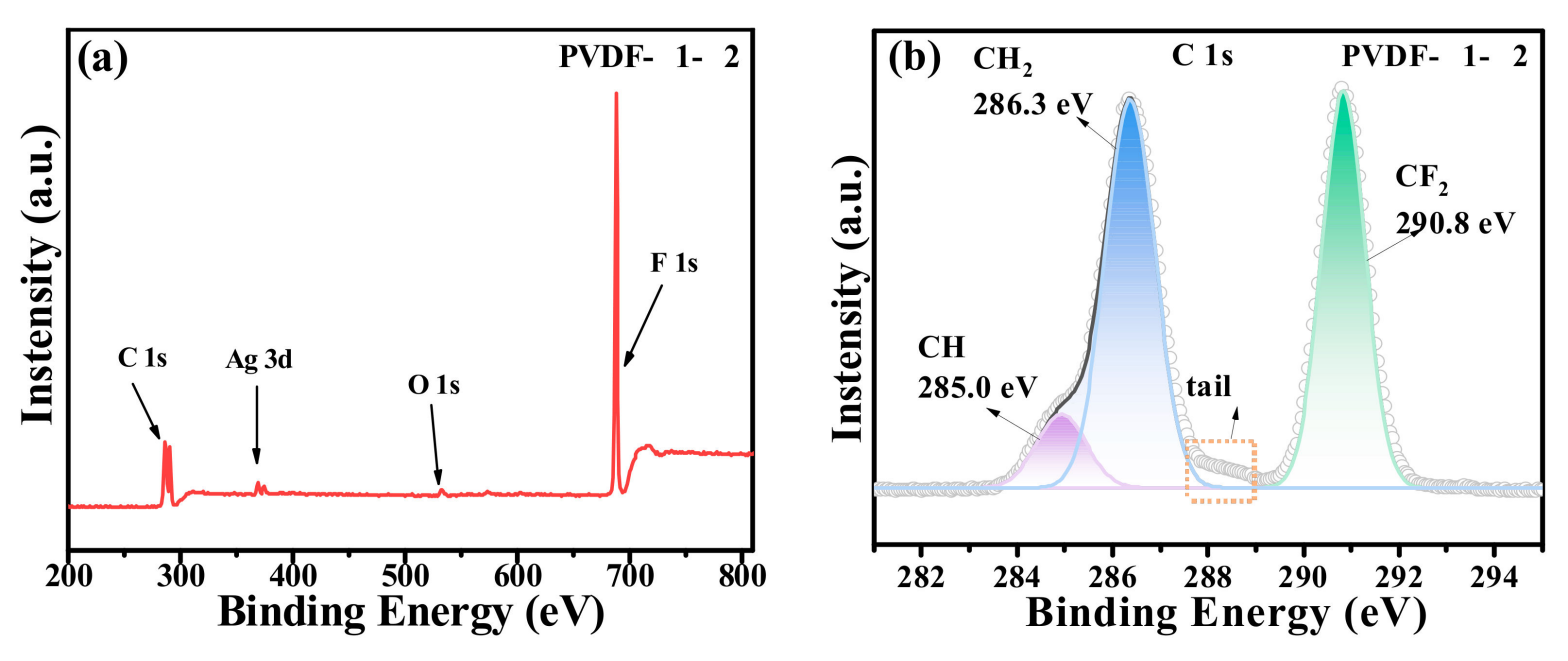

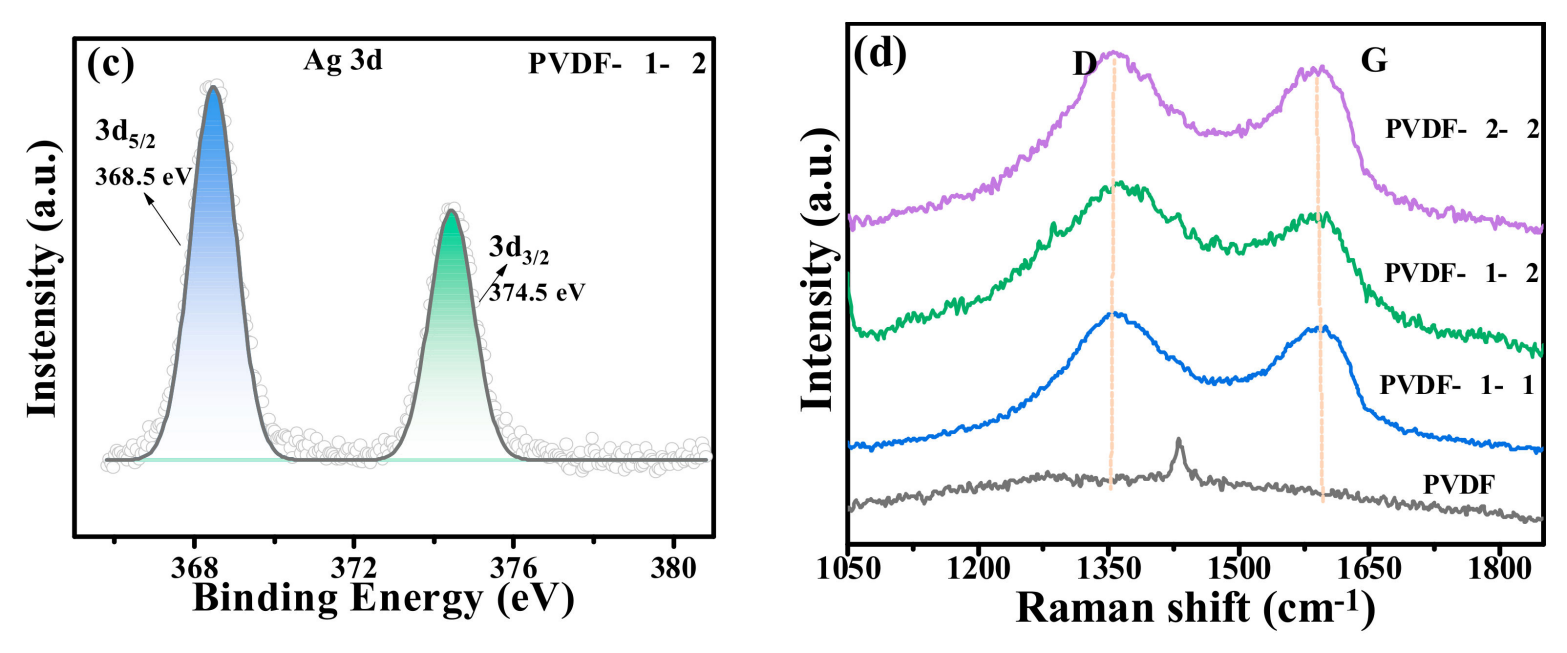
(**a**) XPS survey spectra; (**b**) high−resolution C 1s and (**c**) Ag 3d spectra of PVDF-1-2 fibrous membrane; (**d**) Raman spectra of pristine PVDF and PVDF−based hybrid membranes.

XPS measurement was conducted to analyze the elemental composition and chemical states of the PVDF-1-2 for identifying further the incorporation of GO-Ag into PVDF fibrous membrane. Figure 3a shows the XPS survey spectrum of the PVDF-1-2 membrane. It is easily observed that there are two strong peaks centered at around 290 and 688 eV along with two weak peaks centered at 368 and 532 eV; these peaks correspond to C 1s, Ag 3d, O 1s, and F 1s [42], respectively. The presence of Ag 3d and O 1s peaks confirmed that Ag and GO were successfully incorporated into the PVDF matrix. The high-resolution XPS spectra of C 1s (Figure 3b) could be deconvoluted into two strong peaks at 286.3 and 290.8 eV along with a weak peak at 285.0 eV, which are attributed to the -CH_2_-, -CF_2_-, and -CH- bond [43,44], respectively. In addition to the three peaks, there is a tail peak at around 288.0 eV, which is associated with the -C=O, -COOH, and -CO- bonds present in GO [45,46]. Figure 3c shows the high-resolution spectrum of Ag 3d; the two wide and strong peaks at 368.5 and 374.5 eV correspond to Ag 3d_5/2_ and Ag 3d_3/2_ [47]. It is obvious that the binding energy difference between Ag 3d_5/2_ and Ag 3d_3/2_ is 6 eV belonging to Ag(0) peaks instead of Ag^+^ [48,49], which indicates that Ag is present as metal ions in the hybrid membrane and acted as the catalyst in the catalytic tests.

Finally, the electrospun PVDF-based fibrous membranes were further characterized by Raman spectroscopy, and the spectra are displayed in Figure 3d. Apparently, the pristine PVDF membrane exhibits only a very weak peak at 1432 cm^−1^ without the characteristic peaks of carbon materials. On the contrary, the typical characteristic D and G bands of carbon located at 1350 and 1590 cm^−1^ can be observed in the PVDF-1-1, PVDF-1-2, and PVDF-2-2 fibrous membranes. The D band is attributed to the disorder of edges or structural defects of the GO nanosheets, while the G band is associated with the E_2g_ symmetry and in-plane vibration of sp^2^ carbon atoms [50,51,52]. The Raman results confirmed again that GO had been successfully embedded into the PVDF hybrid membranes.

### 2.2. Catalytic Performance of PVDF@GO-Ag Hybrid Membranes

It is challenging to remove phenolic compounds from wastewater, but reduction of them is one of the effective methods. For the removal of 4-NP, it is worthwhile to reduce 4-NP in the presence of NaBH_4_ as reducing agent, for this reaction is environmentally friendly. However, the reduction of 4-NP with NaBH_4_ is thermodynamically favorable, based on the redox pair potential of 4-NP/4-AP (E^0^ = −0.76 V) and NaBH_4_ (−1.33 V), and the efficient reduction requires a catalyst owing to the high energy barrier for electron transfer from BH_4_^−^ to 4-NP [53]. Thus, the as-electrospun PVDF@GO-Ag hybrid membranes were used as the catalysts to catalytically reduce 4-NP into 4-AP, and the catalytic efficiency was examined in a batch reactor, as shown in Figure S1.

**Figure 4:**
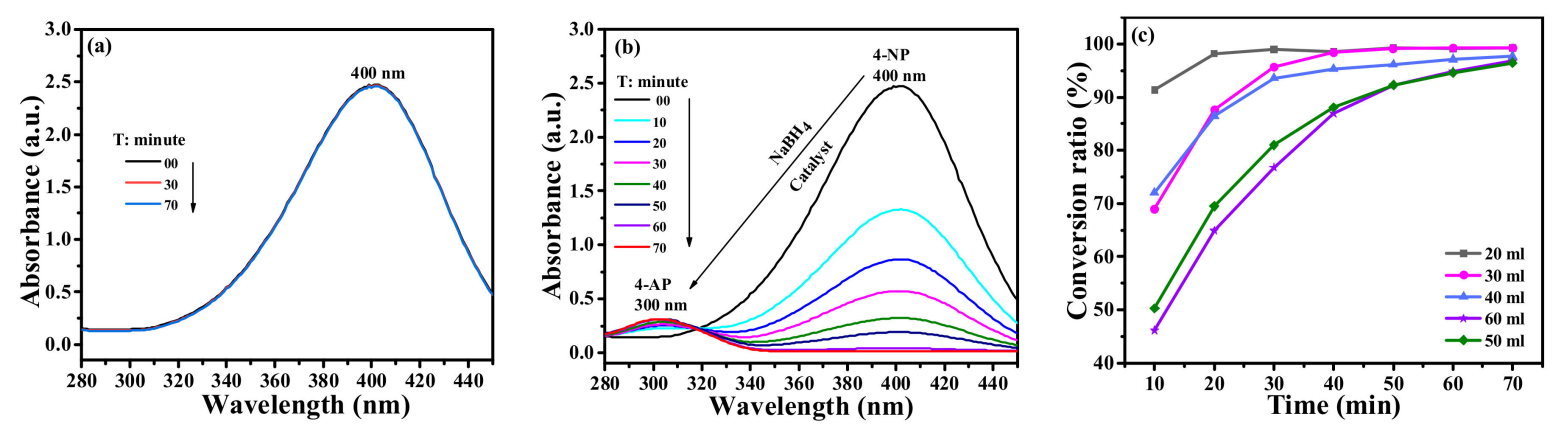
UV-Vis spectra of the reduction of 4-NP catalyzed by (**a**) PVDF and (**b**) PVDF-1-2. Reaction conditions: weight of membrane = 40 mg; [4-NP] = 0.144 mM; [NaBH_4_] = 0.127 M; volume of solution: 50 mL; room temperature; (**c**) conversion ratio of 4-NP under different initial amounts of 4-NP (20, 30, 40, 50, 60 mL) catalyzed by PVDF-1-2.

The quantitative reaction process of 4-NP reduction was monitored with respect to time by UV-Vis spectroscopy. It is well-established that the aqueous solution of 4-NP-NaBH_4_ exhibits a maximum absorption wavelength of approximately 400 nm, which is attributed to the formation of 4-NP^−^ [54], and the solution appears bright yellow with the addition of NaBH_4_ (Figure S1a). Furthermore, the maximum absorption wavelength of the product 4-AP was observed to be at approximately 300 nm. As Figure 4a shows, the PVDF-guided reaction had no effect on the UV-Vis absorption spectrum of the solution, indicating that PVDF itself is not catalytically active. In contrast, the reaction guided by the PVDF-1-2 composite membrane shown in Figure 4b resulted in the disappearance of the characteristic peak at 400 nm over time, while a new characteristic peak 300 nm in wavelength emerged, indicating that 4-NP was reduced to 4-AP. After about 70 min, the reaction solution became colorless (Figure S1b), which demonstrated that the reaction was essentially completed and 4-NP had been reduced to 4-AP. The experimental results demonstrated that the PVDF-based hybrid membranes exhibited strong ability to catalyze the reduction of 4-NP. As illustrated in Figure 4c, the complete reduction of 4-NP can be attained within 20 min, when the initial amount of 4-NP (0.144 mM) was decreased to 20 mL. Even if the initial 4-NP was increased to 60 mL, the reaction process was close to that under 50 mL. In the other tests, 50 mL 4-NP (0.144 mM) was chosen to evaluate the catalytic performance of the hybrid catalytic membrane. 

**Figure 5:**
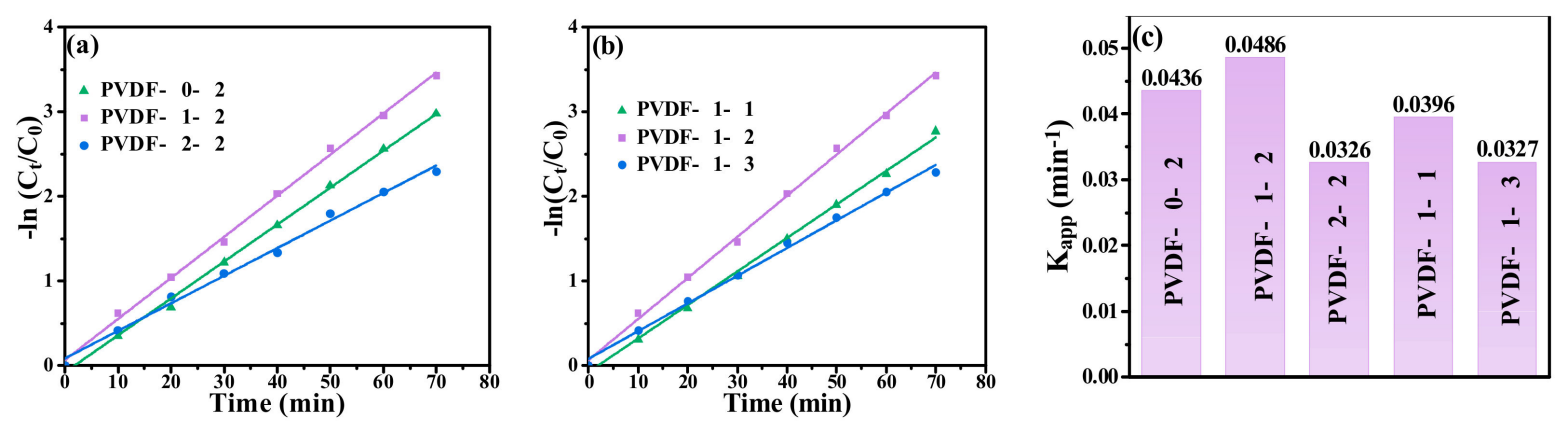
(**a**,**b**) Pseudo-first-order kinetic fitting graphs of catalytic reduction of 4-NP by PVDF-based hybrid membranes; (**c**) comparison of K_app_ for PVDF-based hybrid membranes. Reaction conditions: weight of membrane = 40 mg; [4-NP] = 0.144 mM; [NaBH_4_] = 0.127 M; volume of solution: 50 mL; room temperature.

The reaction can be considered as pseudo-first-order because of the excess addition of NaBH_4_, and the apparent rate constant (K_app_, min^−1^) for the reduction of 4-NP was calculated according to the following kinetic equation:

(1) $$-\ln\left(\frac{C_{t}}{C_{0}}\right)=-\ln\left(\frac{A_{t}}{A_{0}}\right)=K_{\mathrm{app}}t$$

where C_0_ and C_t_ represent the initial concentration of 4-NP and that at time t, and A_0_ and A_t_ are the absorbance intensities at λ = 400 nm at the initial time and t, respectively. Catalytic performance tests were conducted on composite membranes with varying silver loadings and different graphene oxide loadings. As illustrated in Figure 5a,b, the linear correlation between ln(C_t_/C_0_) and the corresponding reaction time demonstrates that the catalytic reaction system with different composite membranes adheres to the pseudo-first-order kinetics. Consequently, the apparent rate constant, K_app_, can be derived directly from the linear fitting results. It is notable in Figure 5c that the catalytic abilities of GO-Ag membranes varied with different mass ratios. Among these membranes, the catalytic reaction rate constant of PVDF-1-2 was 0.0486 min^−1^, which was higher than the K_app_ values of other membranes. Furthermore, from Figure 5a,c, it can be analyzed that compared with the PVDF-0-2 doped with Ag NPs only, the Kapp value increased from 0.0436 min^−1^ to 0.0486 min^−1^ after the addition of 1 wt% of GO, whereas the Kapp value decreased to 0.0396 min^−1^ after the addition of 2 wt% of GO. This can be explained by the effect of the addition of an appropriate amount of GO in reducing the aggregation of silver, which consequently enhances the catalytic performance of the hybrid membrane. Conversely, an excess of GO will surround the Ag and obscure some active sites, resulting in a reduction in the catalytic activity of the composite membrane. An in-depth analysis of Figure 5b,c shows that a reduction in the Ag mass to 1 wt% or an increase to 3 wt% results in a decline in the catalytic efficiency when compared with PVDF-1-2. This decrease in activity may be attributed to a reduction in the active site as the mass of Ag decreases, leading to a reduction in the K_app_ value. An increase in the concentration of Ag by 3 wt% results in the agglomeration of silver nanoparticles due to the limited number of GO anchor sites [55], which subsequently leads to a reduction in the catalytic activity of the hybrid membrane. 

**Figure 6:**
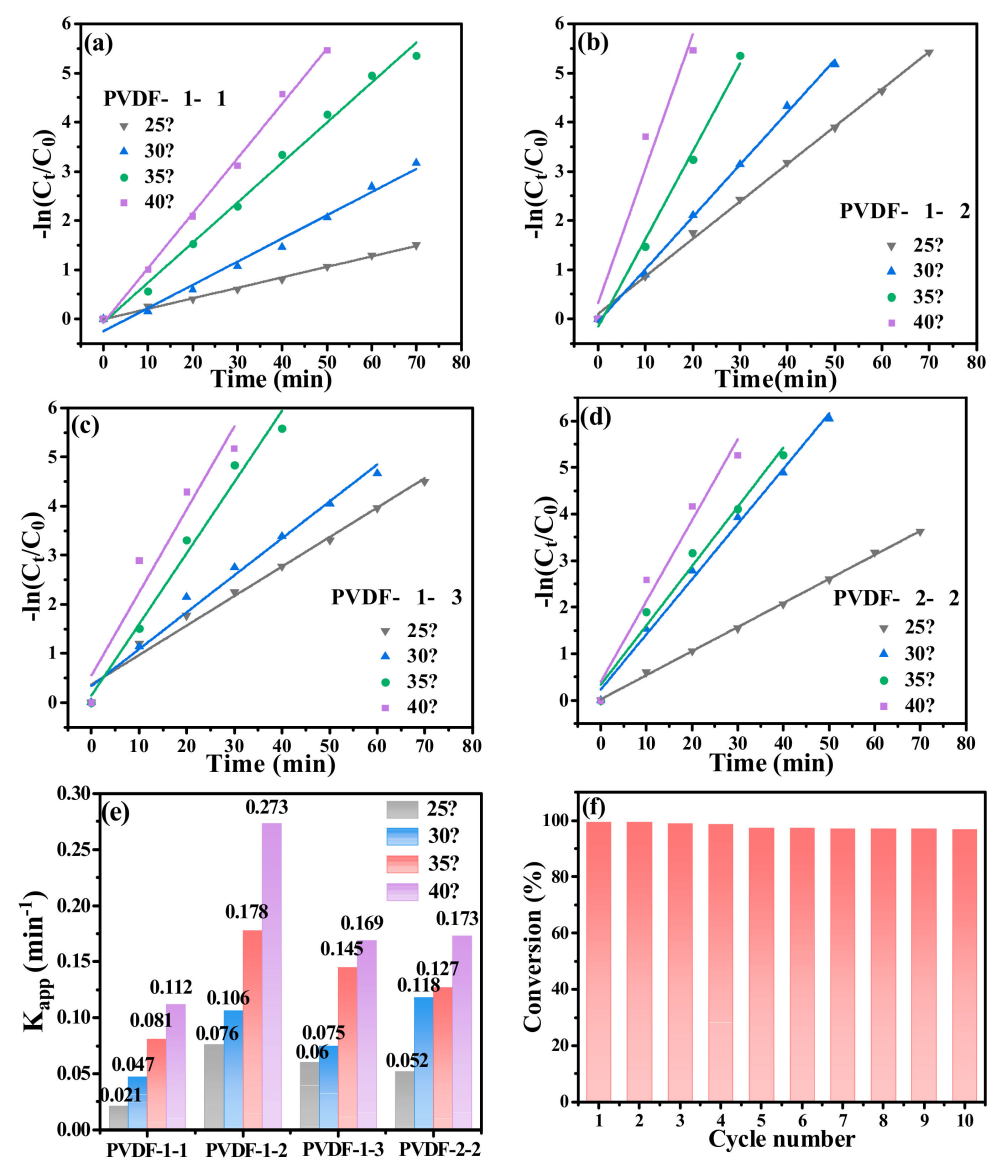
(**a**–**d**) Pseudo-first-order kinetic fitting graphs of catalytic reduction of 4-NP by PVDF composites membrane at the different temperatures. (**e**) Comparison of K_app_ for PVDF composites membrane at different temperature; (**f**) catalytic reduction of 4-NP by PVDF-1-2 in 10 cycles.

To investigate the impact of temperature on the catalytic reaction rate, the PVDF-based hybrid membranes were subjected to the reaction system at temperatures between 25 and 40 °C. As displayed in Figure 6a–d, the kinetic fitting data plots of the 4-NP reduction reaction system guided by each catalytic membrane at different temperatures are presented. It is evident that the reaction rate of each reaction system was markedly accelerated with an increase in temperature. As compared in Figure 6e, for each 10 °C increase in temperature, the K_app_ increases by a factor of 2–4 times the original value, in accordance with the van’t Hoff rule. It is notable that the reaction rate of PVDF-1-2 was the highest under all temperature conditions.

For pure noble metal nano-catalysts, poor reusability is the main bottleneck that prevent them from being used in practical applications [56,57]. The PVDF-based hybrid membranes prepared in this work are dedicated to solving this appealing problem. Therefore, the recyclability test of the membrane is essential. Ten consecutive catalytic reaction experiments were carried out on PVDF-1-2 at room temperature to test the stability of the composite membranes, and the experimental results are shown in Figure 6f. Satisfactorily, the conversion can still be maintained above 95% in 10 cycle tests. In comparison to previous reports about GO-Ag nanocomposites indicating that the conversion of 4-NP was less than 80% after 6–7 cycles of use [28,58], PVDF-1-2 was observed to demonstrate enhanced stability, exhibiting performance characteristics comparable to those of fixed-bed systems [59]. This is attributed to two aspects: (1) As described in the DFT study section, Ag^+^ reacts with hydroxyl (-OH) and epoxy groups (-O-) of GO, and Ag is firmly bonded to GO through covalent bonding, which makes the active component GO-Ag less susceptible to decomposition in cyclic testing. (2) Due to the strong and specific interaction between the carbonyl group (C=O) of GO and the fluorine group (-CF_2_-) in PVDF [40], GO is firmly attached to PVDF, which makes the active component GO-Ag difficult to dislodge in the cyclic tests. This indicates that the vast majority of GO-Ag can be stably loaded on the composite membrane without being washed out, while the aggregation of nanoparticles is greatly reduced. It is worth mentioning that the prepared membranes can be easily and quickly separated after the completion of the reaction, which solves the problem of difficult recycling of noble metal nano-catalysts.

### 2.3. Investigation of the Effects of Ag and GO Ratios on Catalytic Performance via DFT

**Figure 7:**
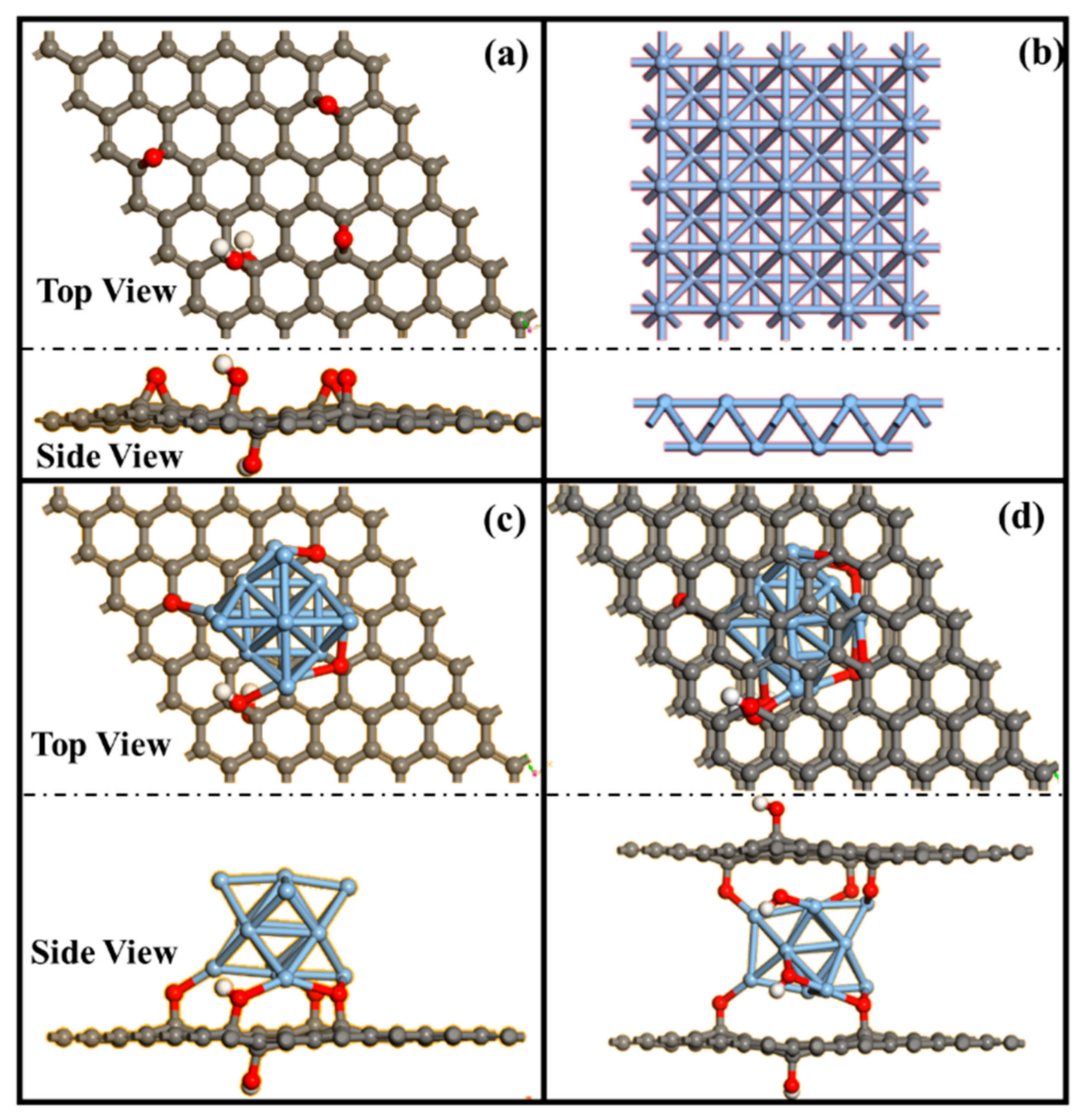
Top and side views of optimized structures. (**a**) GO; (**b**) Ag-B; (**c**) GO-Ag; (**d**) GO-Ag-GO.

A significant change in the catalytic activity of PVDF-based hybrid membranes was observed following a change in the Ag to GO mass ratio. Density functional theory calculations were performed to further understand this phenomenon. It is well known that GO oxygen-containing functional groups include hydroxyl (-OH), epoxy (-O-), carboxyl (-COOH), and carbonyl (-C=O) [60]. Among these, the coexistence of -OH and -O- is often regarded as the metal nanoparticle anchor localization site [61]. Based on this, the present work constructs a theoretical model of GO as shown in Figure 7a, which contains 72 carbon atoms and 3 epoxy and 2 hydroxyl groups. The geometrically optimized Ag (1 1 1) [62] surface model shown in Figure 7b was used to represent the surface of bulk Ag when there was an excess of Ag or a deficiency of GO, which was denoted as Ag-B. Figure S2 depicts a model of Ag nanoclusters when there was a moderate amount of Ag, which contains 14 Ag and is denoted as Ag NC. The optimized structure of the GO-Ag is shown in Figure 7c, which was formed after the Ag NC was anchored to the GO. It can be observed that the Ag NC binds by covalent bonding with oxygen-containing functional groups on the GO surface, which is consistent with other related reports [61,63,64]. In the event of an excess of GO or an insufficient quantity of Ag, a portion of the Ag is covered by the excess GO, resulting in the formation of the GO-Ag-GO structure, as illustrated in Figure 7d.

**Figure 8:**
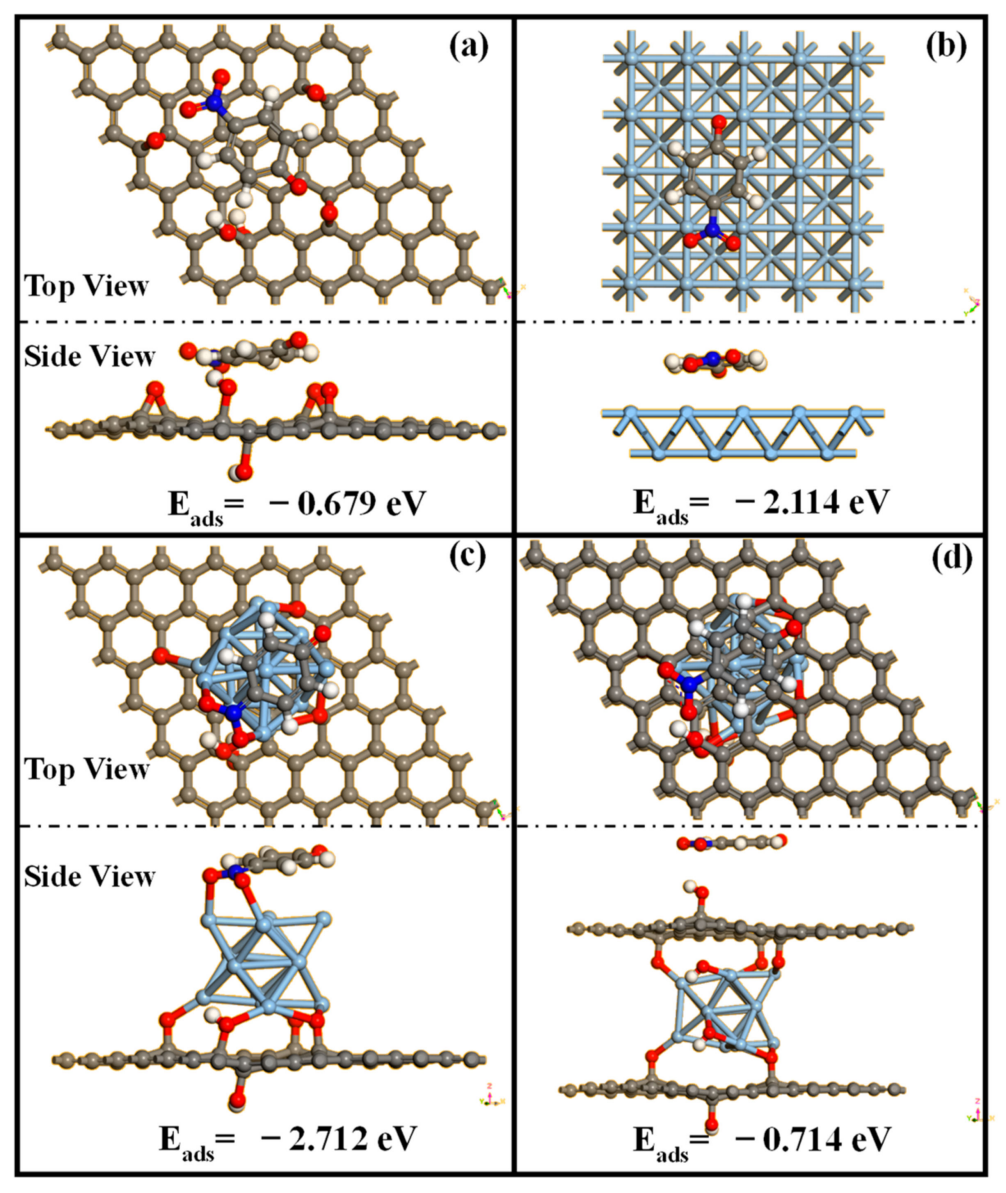
The optimized structures and corresponding adsorption energy for adsorption of 4-NP- on (**a**) GO; (**b**) Ag-B; (**c**) GO-Ag; (**d**) GO-Ag-GO.

Previous studies have indicated that the adsorption of 4-NP^−^ on the catalyst surface represents a pivotal step in the reduction reaction [65,66]. Consequently, the magnitude of the adsorption energy (E_ads_) of 4-NP^−^ by different catalyst models is regarded as a key criterion for evaluating the activity of the catalysts at the molecular level [51]. The adsorption of 4-NP^−^ by the four catalyst models and the corresponding E_ads_ are presented in Figure 8. The adsorption of 4-NP^−^ by GO without Ag loading was found to be relatively weak, with an adsorption energy of only −0.679 eV. In contrast, both Ag-B and GO-Ag exhibited considerable adsorption energies. When Ag was anchored on top of GO in the form of nanoclusters, the E_ads_ was found to be −2.712 eV for 4-NP^−^, indicating that GO-Ag had the strongest adsorption capacity for 4-NP^−^ and possessed the highest catalytic activity. It is noteworthy that the nitro oxygen atom in 4-NP^−^ forms a coordination bond with the Ag atom, as illustrated in Figure 8c. This is conducive to the subsequent hydrodeoxygenation reaction. When silver agglomerates, as illustrated in Figure 8b, the E_ads_ to 4-NP^−^ decrease in size and the adsorption capacity is weakened. In contrast, 4-NP^−^ does not form a chemical bond with the Ag (1 1 1) surface, which is unfavorable for the reaction. Upon the masking of the Ag nanoclusters by GO, the adsorption capacity for 4-NP^−^ was subsequently decreased. The weak interaction of GO-Ag-GO was mainly attributed to the “π-π” conjugation between the GO plane and the 4-NP^−^ benzene ring.

In conclusion, the active components of GO-Ag nanocomposites in PVDF-0-2, PVDF-1-2, and PVDF-2-2 correspond to the models Ag-B, GO-Ag, and GO-Ag-GO, respectively. It can be readily discerned that the ordering of K_app_ values of the different membranes is consistent with the ordering of the adsorption energies of the corresponding models for 4-NP^−^ as follows: K_app_(PVDF-1-2) > K_app_(PVDF-0-2) > K_app_(PVDF-2-2); E_ads_(GO-Ag) > E_ads_(Ag-B) > E_ads_(GO-Ag-GO). This correspondence is more clearly illustrated in Figure S3, which suggests that the activity of the GO-Ag nanocomposites in the hybrid membranes is the primary factor influencing their catalytic performance. Similarly, the catalytic performance of PVDF-1-1 was inferior to that of PVDF-1-2 due to a reduction in active sites, while PVDF-1-3 exhibited agglomeration due to an excess of Ag. This is evidenced by the conversion of the model GO-Ag to Ag-B, which resulted in a decline in catalytic performance.

### 2.4. Possible Catalytic Mechanism

**Figure 9:**
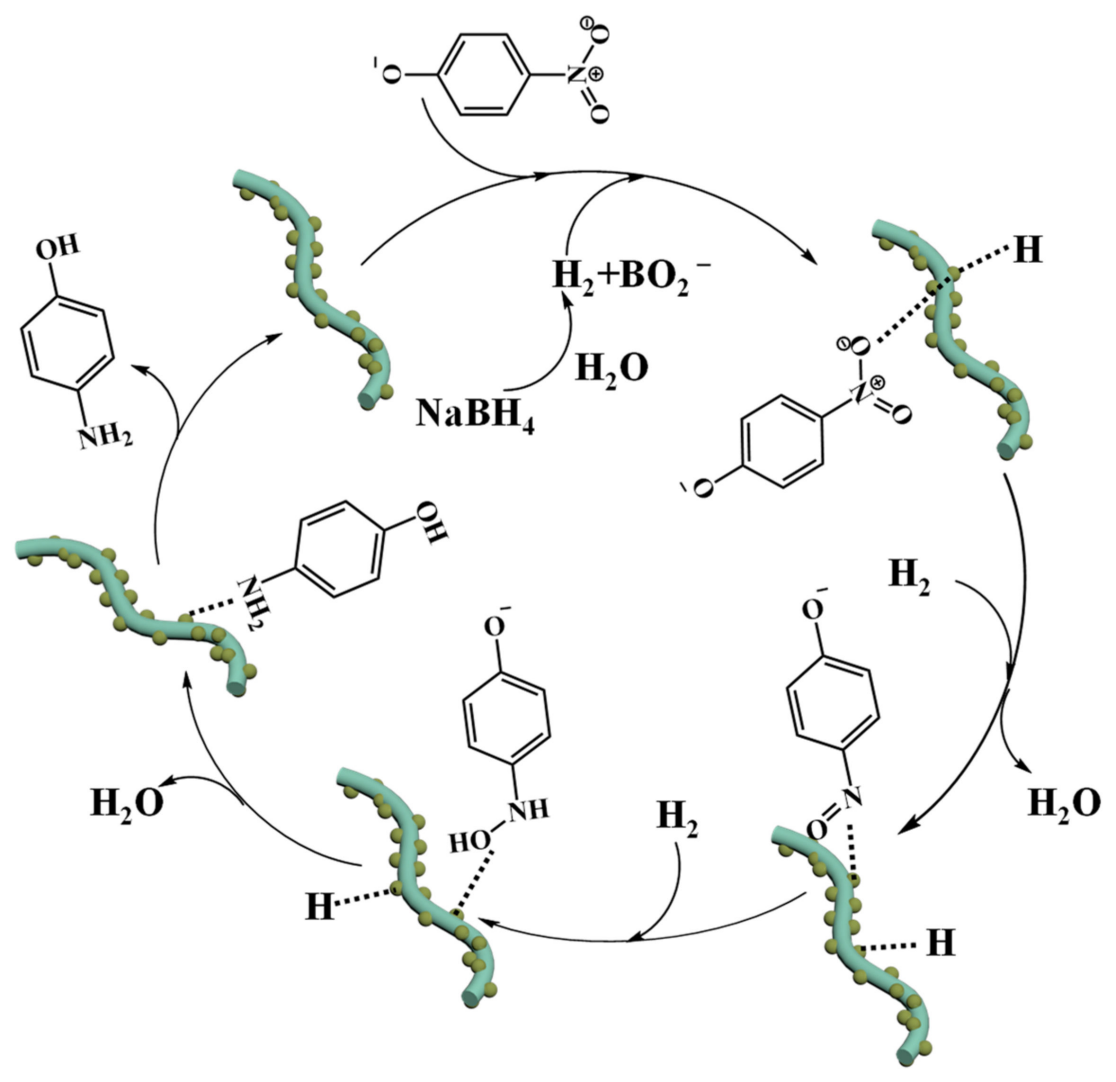
Possible catalytic mechanism for the reduction of 4-NP to 4-AP over PVDF@GO-Ag using NaBH_4_ as hydrogen donor in aqueous solution.

The catalytic reduction of 4-NP to 4-AP in aqueous solution by NaBH_4_ using Ag nanoclusters as active sites has been extensively studied. With reference to analogous catalytic systems [67,68,69,70,71], a possible mechanism for the reduction of 4-NP to 4-AP in aqueous solution by NaBH_4_ catalyzed by PVDF-based hybrid membranes is proposed here (Figure 9): Firstly, the hydrolysis of NaBH_4_ in aqueous solution generates H_2_ and BO_2_^−^. It is important to note that the hydrolysis of generated BO_2_^−^ makes the solution alkaline, and meanwhile, the phenolic hydroxyl group of 4-NP dehydrogenates in an alkaline environment to generate 4-NP^−^. Secondly, H_2_ and 4-NP were adsorbed on the surface of GO-Ag, resulting in the generation of active H. Thirdly, 4-NP^−^ was hydrogenated and dehydrated to form 4-nitrosophenol ion (4-NO^−^). Fourthly, 4-NO^−^ was hydrogenated to form N-(4-hydroxyphenyl)-hydroxylamine ion, which was subsequently dehydrated and then rehydrogenated to form 4-AP. Finally, 4-AP was desorbed.

## 3. Materials and Methods

### 3.1. Chemicals and Materials

The graphite flakes were provided by Sichuan Tianhui Development Co., Ltd. (Luzhou, China). 4-nitrophenol, Poly(vinylidene fluoride) (PVDF) pellets, and sodium borohydride (98%) were supplied by Wuhan Geo Chemical Technology Co., Ltd. (Wuhan, China). Other chemical reagents, such as silver nitrate (AgNO_3_), sulfuric acid (H_2_SO_4_), hydrogen peroxide (H_2_O_2_), hydrochloric acid (HCl), potassium permanganate (KMnO_4_), N, N-dimethylformamide (DMF), and phosphoric acid (H_3_PO_4_), were all analytical reagents. All chemicals were used as received without further purification.

### 3.2. Synthesis of Graphene Oxide (GO)

Graphite oxide (GO) was fabricated based on the modified Hummer’s method [72]. Typically, the mixture of graphite (3.0 g) and KMnO_4_ (18.0 g) was slowly added into the mixed solution containing concentrated H_2_SO_4_ and phosphoric at a ratio of 9:1 (360 and 40 mL); during this reaction, some heat will be released. Then, they were placed in a thermostat water bath and heated to 50 °C, fully stirring for reaction at this temperature for 12 h. Subsequently, the product was cooled to room temperature and transferred to an ice bath, and 3~5 mL 30% H_2_O_2_ was slowly dropped to the obtained solution under continuous stirring until the solution became golden yellow. Next, the yellow solution was centrifuged to remove the supernatant and the residue was rinsed with dilute HCl and ionized water repeatedly until the pH value of the supernatant was 7. Finally, the resulting product was frozen with liquid nitrogen, dried in a vacuum freeze dryer for about 72 h, and stored in refrigerator for later use.

### 3.3. Preparation of Ag-GO Nanocomposites

The catalytic active component (Ag-GO) was prepared by an in situ reduction method. Firstly, a certain amount of GO (20 mg) was dispersed in DMF under ultra-sonication for 1 h at room temperature to form a stable GO suspension. Then, a desired quantity of AgNO_3_ (silver precursor) was added into the suspension under sonication for another 30 min. Subsequently, the suspension was heated to 60 °C under magnetic stirring and kept at this temperature for 12 h in a water bath. During this process, DMF acted as dual role of solvent and reducing agent; the obtained Ag-GO suspension can be kept homogeneous and very stable by judiciously controlling the reduction temperature and time, and no visible dark precipitate formed. To investigate the effects of silver and GO amount on the catalytic performance, Ag-GO nanocomposites were prepared with different AgNO_3_/GO mass ratios. As a control, pure Ag NPs were prepared by reduction of AgNO_3_ by DMF in the absence of GO.

### 3.4. Electrospun and Synthesis Mechanism of PVDF@GO-Ag Hybrid Membranes

The PVDF-based membranes were fabricated using a direct electrospinning method. To prepare pristine PVDF membrane, PVDF pellets were dissolved in DMF with a mass ratio of 20:80, and magnetically stirred for 5 h at 50 °C. For the preparation of PVDF@Ag and PVDF@GO-Ag nanocomposites membranes, 20 wt% PVDF (in DMF) solution was first prepared, then a given amount of Ag/DMF or GO-Ag/DMF suspensions was added into the PVDF solution and magnetically stirred for 5 h at 50 °C until well blended. For the PVDF-Ag-GO system, GO content was controlled at 0 to 2 wt% while the Ag NPs were kept at 1 to 3 wt% relative to the weight of PVDF. The prepared suspension was loaded into a syringe with stainless steel needle tip and then placed onto an electrospinning setup. During the electrospinning process, a voltage of 20 kV was applied, the working distance between the needle tip and the collector was maintained at 15 cm, and the flow rate was kept at 0.5 mL/h. A grounded and aluminum-foil-wrapped rotating mandrel was employed as the collector. Finally, the obtained electrospun fibrous membranes were dried in a vacuum at 60 °C to eliminate any residual solvents. The membranes obtained with different compositions were denoted as PVDF-X-Y, where X, Y represent the mass ratios of GO and Ag, respectively, with respect to PVDF. For example, the membrane comprising 1 wt% GO with 2 wt% Ag nanocomposites was denoted as PVDF-1-2.

**Scheme 1:**
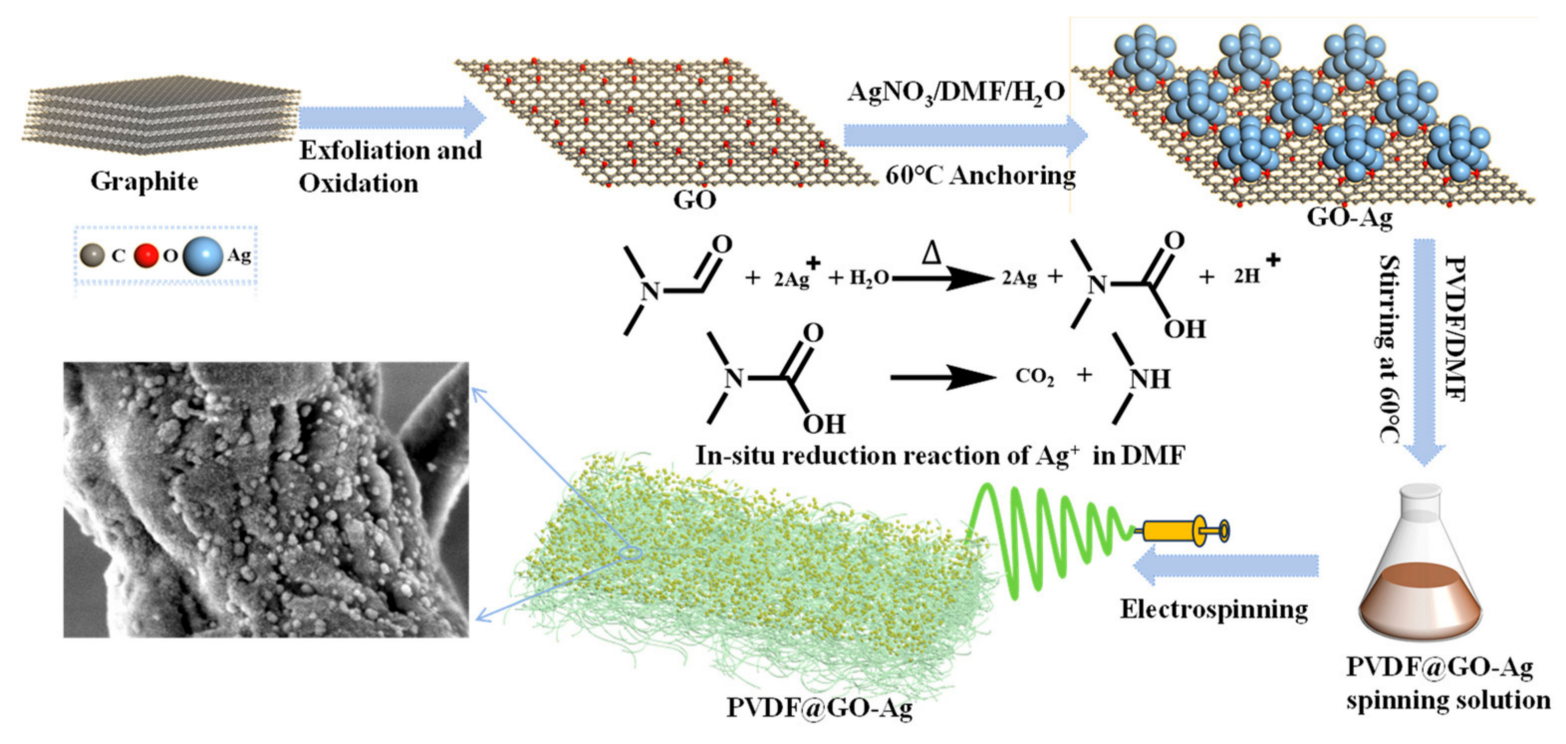
The synthesis mechanism of nanocomposites GO-Ag and PVDF@GO-Ag hybrid membranes.

Scheme 1 illustrates the synthesis mechanism of the Ag-GO suspension and electrospun process of the hybrid membrane. In this route, the Ag NPs were obtained using AgNO_3_ as precursor and DMF as reducing agent by an in situ synthesis method. As is well known, DMF is a common organic solvent for various processes, and here it was used to dissolve PVDF. Furthermore, DMF can be an active reducing agent under proper conditions. Liz-Marza’n et al. [73] confirmed that DMF has the ability to reduce Ag^+^ to zerovalent Ag at room temperature without any external agent; the proposed reaction is shown in Scheme 1. In addition, Ag NPs decorated on GO can enhance their properties [74,75,76], and it is crucial to disperse Ag-GO hybrid sheets uniformly in the polymer matrix when preparing hybrid membranes. Here, the GO was directly dispersed in DMF to form a GO suspension, followed by the addition of AgNO_3_ solution under sonication; the Ag-GO suspension was obtained and is shown in Figure S4. Then, the polymer matrix (PVDF) was added into the suspension and electrospun into PVDF@GO-Ag membrane.

### 3.5. Characterizations of PVDF-Based Membranes

The morphology and structure of the as-prepared membranes were observed with field emission scanning electron microscope (FE-SEM) (FEI USA, Inc. Nova NanoSem 450 Hillsboro, OR, USA); SEM elemental mapping of samples was also recorded on this instrument with Brucker Quantax 400 X-ray (Bruker GmbH, Ettlingen, Germany). Total reflection Fourier transform infrared (FTIR-ATR, Thermo Nicolet iZ10, Appleton, WI, USA) was employed to analyze the functional groups and chemical compositions on the surface of the PVDF membrane. Raman spectra were recorded on Lab Ram HR800 Raman spectrometer (HORIBA Jobin Yvon, Palaiseau, France). X-ray photoelectron spectroscopy (XPS) measurements were carried out on ESCALAB250 spectrometer (Thermo Fisher Scientific, Waltham, MA, USA) with an Al radiation source. The contact angle was used to evaluate the wettability and hydrophilicity, and the contact angle of the surface of the PVDF modified membrane was measured at room temperature by using a contact angle measuring instrument (JC2000A Shanghai JianDuan Photoelectricity Technology Co., Ltd, Shanghai, China). UV-Vis spectra were recorded on a TU-1810 (Puxi General Instrument Co., Ltd., Beijing, China) spectrophotometer.

### 3.6. Catalytic Performance Test

The as-fabricated PVDF-based hybrid membranes were used to catalytically reduce of 4-NP to 4-AP using NaBH_4_ as the reducing agent. Briefly, 20 mg 4-NP was dissolved into 50 mL DI water in a beaker, 0.24 g NaBH_4_ was added into the 4-NP solution, and 40 mg the as-prepared membrane was placed into the mixed solution under slow magnetic stirring at room temperature. After every 10 min, 2 mL of each sample was taken out from the reaction solution without any further purification, then the absorption spectrum of each sample was recorded by UV-Vis spectroscopy in the wavelength range of 280–570 nm to monitor the reaction progress. The reusability of the composite catalytic membrane was examined for several consecutive cycles. After completion of each catalytic reduction, the membrane was taken out easily from the reaction system with tweezers. Then, it was soaked in ethanol solution and rinsed with DI water several times, followed by drying in a vacuum oven at 90 °C overnight for further catalytic reduction cycles. The time-dependent conversion ratio of 4-NP to 4-AP was evaluated using Equation (2):

(2) $$\mathrm{conversion}\;\left(\%\right)=\left(1-\frac{A_{t}}{A_{0}}\right)\times 100\%$$

where A_t_ and A_0_ are the absorbance of the solution at time t and the initial time, respectively.

### 3.7. Computational Methods

Density functional theory calculation were performed by the DMol^3^ package (Materials Studio 2020) [77] using Perdew–Burke–Ernzerh exchange–correlation functional [78] of generalized gradient approximation (GGA-PBE) with Grimme’s default DFT-D parameters. The double numerical plus polarization function (DNP) basis set were used. The integration of the Brillouin zone was sampled by 4 × 4 × 1 *k*-points within the Monkhorst–Pack scheme. A graphene sheets containing 72 carbon atoms was chosen to construct the GO substrate with a vacuum region of 20 Å to prevent the disturbance between layers. In order to model the surface of aggregated Ag, an Ag (1 1 1) surface was selected, which was then modelled using a 3 × 3 supercell containing 2 layers. Ag nanoclusters with a radius of 3 Å were constructed with 14 silver atoms. It should be noted that all atoms were not fixed during the optimization. The adsorption energies (E_ads_) of different catalysts for 4-NP^−^ were calculated using the following equation:

(3) $$E_{\mathrm{ads}}=E_{\mathrm{cat}+{4-\mathrm{NP}}^{-}\;}-E_{\mathrm{cat}}\;-E_{{4-\mathrm{NP}}^{-}}$$

where $E_{\mathrm{cat}+{4-\mathrm{NP}}^{-}\;}$,$E_{\mathrm{cat}}$, and $E_{{4-\mathrm{NP}}^{-}}$, are the total energy of the catalyst after adsorption of 4-NP^−^, catalyst, and 4-NP^−^, respectively. 

## 4. Conclusions

In conclusion, the poly(vinylidene fluoride)-based hybrid membranes (PVDF@GO-Ag) were successfully loaded with the graphene oxide-silver (GO-Ag) nanocomposites onto the PVDF fibers by electrospinning. A batch reaction study of the PVDF@GO-Ag hybrid membranes using the hydrogenation of 4-NP as a model reaction yielded 100% conversion to 4-AP within 70 min. Furthermore, the impact of the ratio of GO and Ag on the efficacy of catalytic reduction of 4-NP was evaluated. The catalytic reduction of 4-NP by hybrid membranes conformed to pseudo-first-order kinetics. Notably, membranes incorporating 1 wt% GO and 2 wt% Ag, known as PVDF-1-2, demonstrated the highest apparent reaction rate constant, with a value of K_app_ = 0.0486 min^−1^. The catalytic efficiency was lower in nanocomposites with 0 or 2 wt% GO and 2 wt% Ag (PVDF-0-2 and PVDF-2-2) and also when the composition included 1 wt% GO with 1 wt% or 3 wt% Ag (PVDF-1-1 and PVDF-1-3) compared to PVDF-1-2. At the molecular level, s density functional theory (DFT) study demonstrated that an excess of GO would result in coverage of Ag, thereby significantly reducing the adsorption capacity of GO-Ag nanocomposites for 4-NP. Conversely, an excess of Ag would lead to clustering, which would in turn diminish the catalytic activity of GO-Ag nanocomposites. The maximal adsorption capacity for 4-NP was observed for GO-Ag when Ag was anchored onto GO in the form of nanoclusters. Moreover, the study demonstrated that an increase in temperature significantly accelerated the reaction rate, in accordance with the van’t Hoff rule. Importantly, PVDF-1-2 displays remarkable stability when subjected to 10 catalytic cycles. This property effectively addresses the significant challenges associated with the practical application of nanocatalysts. The PVDF@GO-Ag membranes offer an effective and eco-friendly solution for the treatment of 4-NP in wastewater, combining high catalytic performance, stability, and ease of recycling.

## Data Availability

The original contributions presented in the study are included in the article/Supplementary Material; further inquiries can be directed to the corresponding authors.

## Supplementary Materials

The following supporting information can be downloaded at: https://www.mdpi.com/article/10.3390/molecules29163930/s1, Figure S1: Photograph of 4-NP/NaBH_4_/PVDF-1-2 system (a) before and (b) after reaction; Figure S2: Optimized structure of Ag nanocluster; Figure S3: Plot of Kapp values of different component hybrid membranes versus corresponding models’ Eads for 4-NP−; Figure S4: Photographs of Ag-GO suspension.
