# Supplementary material for: Highly Efficient Electrospun Silver Decorated Graphene Oxide Nanocomposites on Poly(vinylidene fluoride) (PVDF@GO-Ag) Hybrid Membrane for Reduction of 4-Nitrophenol
Source: Molecules. 2024 Aug 20;29(16):3930. doi: 10.3390/molecules29163930 (PMC11357165; doi:10.3390/molecules29163930)
Supplement: Supplementary file 1 [file molecules-29-03930-s001.zip › molecules-3129541-supplementary.pdf]

Supplementary Materials

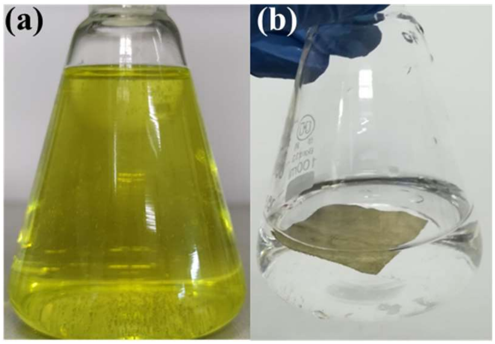

Figure S1. Photograph of 4-NP/$NaBH_4$/PVDF-1-2 system (a) before and (b) after reaction.

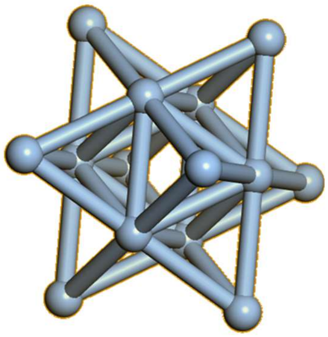

Ag Nanocluster

Figure S2. Optimized structure of Ag Nanocluster.

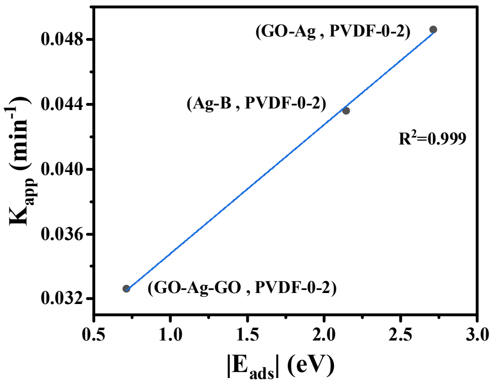

Figure S3. Plot of K_{app} values of different component hybrid membranes versus corresponding models' E_{ads} for 4-NP^-.

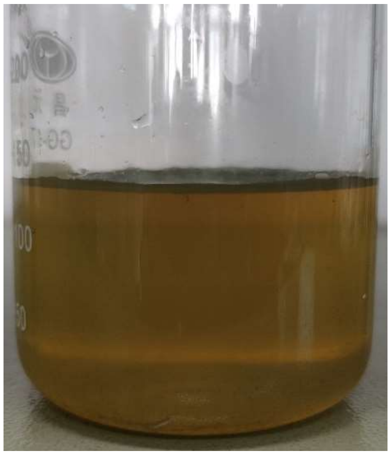

Figure S4. Photographs of Ag-GO suspension.
